# Presbyopia: a pilot investigation of the barriers and benefits of near visual acuity correction among a rural Filipino population

**DOI:** 10.1186/1471-2415-14-9

**Published:** 2014-01-27

**Authors:** Thomas J Wubben, Christopher M Guerrero, Marlo Salum, Gregory S Wolfe, Gerald P Giovannelli, David J Ramsey

**Affiliations:** 1 University of Illinois at Chicago, Chicago IL, USA; 2 Saint Mary's University, Bayombang, Nueva Vizcaya, Philippines; 3 Southern California College of Optometry, Fullerton CA, USA; 4 Rehabilitation Institute of Chicago, Chicago IL, USA; 5 Massachusetts Eye and Ear Infirmary, 243 Charles Street, Boston, MA 02114, USA

**Keywords:** Presbyopia, Quality of life, Survey, Near vision, Stereoacuity, Reading glasses

## Abstract

**Background:**

Presbyopia is the age-related decline in accommodation that diminishes the ability of the eye to focus on near objects. Presbyopia is common and easy to correct; however, many communities lack access to basic eye care. The purpose of this project was to assess the burden of uncorrected presbyopia in a rural Filipino population and to pilot an intervention aimed at increasing access to reading glasses in the community.

**Methods:**

Individuals above the age of 40 who presented to a health outreach in the Philippines were invited to undergo a near vision exam to detect the presence of functional presbyopia and be fitted with ready-made, single-vision glasses. The change in stereoacuity was used as a surrogate measure of functional improvement after near vision correction. A questionnaire was administered to assess this population’s perceived barriers and benefits to correcting near vision.

**Results:**

The average age of the participants was 57 ± 11 years, with 87.6% of participants having an uncorrected near visual acuity of <20/50. Reading glasses improved near vision to 20/40 or better in 77.7% of participants having near-vision impairment (uncorrected near visual acuity of <20/40). Over 75% of participants also showed improvement in stereoacuity. Cost, rather than availability, was perceived to be the greater barrier to the procurement of glasses, and 84% of participants reported that the glasses dispensed would greatly improve their ability to earn a living.

**Conclusions:**

Dispensing ready-made, single-vision glasses is a simple and cost-effective intervention to improve near vision and enhance depth perception. A greater understanding of the barriers and benefits to correcting near vision will inform the design and execution of a sustainable program to correct presbyopia in developing countries.

## Background

Presbyopia is the age-related decline in accommodation that diminishes the ability of the eye to focus on near objects [1]. This process usually becomes perceptible between ages 40 and 50 and accelerates with age, necessitating the application of corrective lenses in order to restore near vision [2]. From a pathophysiologic standpoint, multiple theories have been put forth in an attempt to explain this decline in amplitude of accommodation. Changes in the shape, size, and mechanical characteristics of the lens, as well as the function of the ciliary muscle, have all been described [3,4]. Nonetheless, aging is the most important risk factor for the development of presbyopia.

Presbyopia is common and easy to correct, even when it occurs together with underlying refractive error. Whereas a modest amount of uncorrected myopia may be somewhat protective of the effects of presbyopia, a mild to moderate amount of uncorrected hyperopia tends to accelerate the age at which a decline in near vision is first perceived. While glasses for near vision, commonly referred to as “reading glasses”, provide obvious benefits in a contemporary, literate society, the difficulties caused by uncorrected presbyopia may nonetheless be quite burdensome in areas with lower literacy rates because near visual acuity is required for many activities of daily living besides reading, such as cooking, sewing, and close family interactions [2]. Even though presbyopia is common and easy to correct, in communities that lack access to basic eye care, many needlessly suffer from this visual impairment.

To this end, studies have been performed in developing countries to assess the impact of uncorrected presbyopia [2,5-9]. The results from these studies demonstrate that the prevalence of presbyopia is high in these developing areas while spectacle coverage is low, and that presbyopia is associated with difficulty in near vision-related tasks, some of which may influence one’s ability to earn a living [2,5-9]. Yet, this effect on the quality of life is easily avoided by providing access to affordable spectacles and proper fitting. Moreover, there is increasing recognition that presbyopia significantly adds to the burden of global visual impairment, resulting in presbyopia being included in the most recent World Health Organization action plan for the prevention of avoidable blindness and visual impairment [10].

The current investigation is the first to examine presbyopia in the Philippine Islands, focusing on a rural population in the province of Nueva Vizcaya. This study aims to measure the extent to which the application of single vision reading glasses alone can improve near vision. A simple and cost-effective intervention strategy was developed to rapidly dispense reading glasses to achieve the best corrected, binocular, near visual acuity through a successful collaboration between two international health care organizations. In the process, a pilot survey of the perceived barriers and benefits of correcting near visual acuity, as well as access to the correction of presbyopia, was conducted. A better understanding of these factors will help in the design and execution of a sustainable program to improve near vision in developing countries.

## Methods

Members of the Global Medical Foundation, U.S.A., and Saint Mary’s University School of Health Sciences (Bayombong, Nueva Vizcaya, Philippines) undertook the present project in order to correct presbyopia in an aged Filipino population (>40 years old) in the rural province of Nueva Vizcaya in the Philippine Islands. The villages of Dupax Sur and Bambang were chosen because of their relatively central location and accessibility by passable motorways. A majority of the participants served by the outreach activities are members of several indigenous people groups in the Philippines, protected and legally recognized minorities. In order to comply with the Indigenous Peoples Rights Act of 1997 (Republic Act No. 8371), no ethnographically identifying data was collected out of sensitivity to these indigenous cultural communities. The local provincial authorities were responsible for announcing the availability of the health outreach, which offered a variety of medical and dental services for all ages. Services were also offered to residents of neighboring municipalities who journeyed to the mission sites. One of the services was a mass-screening program aimed at identifying individuals with uncorrected presbyopia and fitting them with reading glasses to correct near vision. A cross-sectional, pilot survey of the perceived barriers and benefits of correcting near visual acuity was administered.

Persons above the age of 40 without bilateral visual impairment or blindness, as determined by both history and a screening exam with a pen light to exclude gross abnormalities, who presented to the health outreach were invited to undergo a near visual acuity exam and be fitted with reading glasses (supplied in part by RestoringVision.org, San Rafael, CA). The near visual acuity exams were conducted by senior nursing students from St. Mary’s University of Health Sciences, who were members of the eye care team, at a standard distance of 40 cm in outdoor illumination, binocularly, using either the Rosenbaum or LEA near vision card, depending on patient preference and/or literacy level. The distance of approximately 40 cm was maintained by a string attached to the bottom of the near visual acuity chart at one end with the other end held taught against the person’s chin. Near visual acuity exams were initially conducted unaided. Persons were then furnished with a pair of reading glasses based on their age, ranging from +1.00 D to +3.50 D [11,12], with the lens power adjusted in quarter to half steps to improve their near visual acuity. A variety of frame sizes were available and fit at the discretion of the examiner; no formal assessment of interpupillary distance was undertaken. As refractive services were beyond the scope of the available resources, no refraction was undertaken, nor was any attempt made to measure each eye individually. Instead, we identified functional presbyopes as those who had impairment of near visual acuity that could be improved at least one line of visual acuity by placing a plus lens in front of either eye.

Functional improvement upon application of reading glasses was assessed by comparing the subject’s score on the RANDOT® stereoacuity test (Stereo Optical Co., Inc., Chicago, IL) with and without the near vision correction provided by reading glasses at a distance of 40 cm. This test was administered by the lead investigator as well as by senior nursing students specifically trained by the lead investigator. This test incorporates simple shapes at two levels of disparity (500 and 250 arc-seconds), pictures of animals arranged at three levels of disparity (400, 200, and 100 arc-seconds), and finally, a series of ten panels that provide a graded disparity between 400 and 20 arc-seconds. Participants who did not demonstrate measurable stereoacuity on a particular portion of the test (simple shapes, animals, or panels) were assigned a value of 1000 arc-seconds (twice the limit of measurable stereoacuity in this study) for the purposes of analysis.

Following the completion of this medical intervention, individuals were invited to participate in a questionnaire. Of note, the intervention was offered regardless of willingness to participate in the survey. Verbal informed consent was obtained and communications took place in Ilocano, a local Filipino dialect prominent in the province of Nueva Vizcaya, or Tagalog or English, both official languages of the Philippines, depending on the participant’s preference and comprehension. Senior nursing students fluent in these languages administered the questionnaire. Participants were not required to answer every question. Some participants may have not understood every question secondary to education level, having a different dialect as their primary language, or not being applicable to their current situation. The questionnaire was based in part on the National Eye Institute’s Visual Function Questionnaire (NEI-VFQ) [13] but modified to reflect issues important to this population. The questionnaire consisted of multiple-choice questions pertaining to basic demographics and the subject’s perceived barriers and benefits to obtaining access to the treatment of presbyopia. Although questionnaires have inherent limitations, this tool provided valuable initial insights into this population’s perceived barriers and benefits and is similar in approach to other studies [5]. The Institutional Review Board at the University of Illinois at Chicago granted exemption for all procedures and protocols, and this study followed the tenets of the Declaration of Helsinki.

All data were analyzed using Microsoft Excel (Redmond, WA) version 12.2.7. All tests of association (t-tests, ANOVA, regression) were considered to be statistically significant at p <0.05.

## Results

More than 600 people voluntarily underwent near visual acuity testing and were fit with reading glasses. Among these, 142 people (24%) agreed to take part in the near vision questionnaire that assessed their perceived barriers and benefits to the treatment of presbyopia. The low response rate may be due in part to the fact that participants preferred to partake in the other services at the health outreach rather than take the questionnaire. The mean age of the participants was 57 ± 11 years, 75.3% were female, and 52.6% had secondary or higher education (Table 1). Further underscoring the poverty amongst the participants, 23% of the participants were unemployed (Table 1). No associations were found between these demographic factors and the participants’ perceived barriers and benefits of correcting near visual acuity.

**Table 1:** Demographic and near visual acuity data for questionnaire participants

		***n***
**Mean age (yr ± SD):**		**142**
	57 ± 11	
**Gender (%):**		142
Male	24.7	
Female	75.3	
**Education (%):**		133
None	3.0	
Primary	44.4	
Secondary	41.3	
College	11.3	
**Occupation (%):**		135
Domestic laborer	31.9	
Farmer	19.3	
Other^a^	25.8	
Unemployed	23.0	
**Uncorrected near visual acuity (n, %):**		137
≥20/40	16, 11.7%	
<20/40-20/63	17, 12.4%	
<20/63-20/200	60, 43.8%	
<20/200-20/400	44, 32.1%	

^a^Other occupations include governement employee, utility worker, driver, teacher, and street vendor.

Among the participants, more than three-quarters of the participants had an uncorrected near visual acuity <20/63 (Table 1). Also, the participants’ uncorrected near visual acuity worsened with age (R = 0.27, p < 0.002), and subsequently, a positive trend was observed between the power of the reading glasses suggested and age, but this trend was not statistically significant (R = 0.11, p = 0.21). Most importantly, though, upon application of reading glasses, 77.7% (94/121) of the participants who had an uncorrected near visual acuity <20/40 corrected to 20/40 or better.

Stereoacuity data was collected from 140 of the 142 participants (Table 2). Ten patients failed to demonstrate perception of stereopsis pre- or post-near correction, despite each demonstrating improvement in near vision upon the application of readers. Among those participants that demonstrated perception of stereopsis, more than three quarters (76.1%, 99/130) showed improvement in tests of stereoacuity upon the application of reading glasses and more than two thirds (67.7%, 88/130) manifested an improvement in arc-seconds of resolution of stereoacuity. The average stereoacuity prior to the application of reading glasses was >400 arc-seconds (463 ± 376 arc-seconds) versus nearly 100 arc-seconds following correction (111 ± 110 arc-seconds) (p < 0.0001). On average, these participants demonstrated a 66% improvement in stereoacuity. Interestingly, a small number of participants (12.3%, 16/130) actually showed a small decline in stereoacuity.

**Table 2:** Perception of stereo post-near correction*

		**Stereoacuity post-near correction**			
	**No stereo perception pre-or post-correction**	**Decline in arc-seconds of resolution**	**No improvement in arc-seconds of resolution**^a^	**Improvement in some tasks**^b^	**Improvement in arc-seconds of resolution**
**Total participants (n = 140)**	10 (7%)	16 (11%)	36 (26%)	99 (71%)	88 (63%)

*n (%); percentages were determined with regards to the total participants from which stereoacuity data was collected.

^a^Includes those who did not perceive stereo pre- or post-near correction.

^b^Includes a portion of those who had a decline in overall arc-seconds of resolution but showed improvement on some tasks but not others.

When asked about their ability to read, 95% (134/141) of the participants responded they were able to read to some degree. Yet, approximately two thirds of the participants stated they had at least a moderate degree of difficulty reading small print, such as that in newspapers. Additionally, when asked about the cost of reading glasses and the availability of an eye doctor as barriers to obtaining reading glasses, participants perceived that cost greatly (46.2%, Table 3) prevented them from obtaining glasses more so than availability (26.0%).

**Table 3:** Questionnaire responses on perceived barriers and benefits to obtaining access to eye care

	**Greatly**	**Moderately**	**A little**	**Not at all**
**Does the cost of reading glasses prevent you from obtaining glasses? (n = 132)**	46.2%	15.2%	34.1%	4.5%
**Does the availability of an eye doctor prevent you from obtaining glasses? (n = 131)**	26.0%	19.1%	36.6%	18.3%

The majority of the participants (68.8%) had a previous pair of reading glasses. Furthermore, 46.2% (36/78) of those who had purchased their previous pair of reading glasses spent >10 USD. The main reason cited for not having a previous pair or reading glasses was lack of money.

The participants who had a previous pair of reading glasses were asked how that pair impacted certain daily tasks (Table 4). Not surprisingly, reading glasses were perceived to have the greatest impact on reading (91.0% of the participants stated reading glasses would be absolutely necessary for accomplishing this task).

**Table 4:** Perceived impact of reading glasses on daily tasks*

**Daily task (n = 67)**	**Absolutely necessary**	**Helpful, but not required**	**Minimally helpful**	**Not at all**	**N/A**
**Reading**	91.0%	6.0%	1.5%	1.5%	0.0%
**Cooking**	17.9%	19.4%	26.9%	32.8%	3.0%
**Sewing**	74.6%	3.0%	4.5%	7.5%	10.4%
**Hobbies**	38.8%	13.4%	16.4%	20.9%	10.4%
**Farming**	17.9%	10.4%	20.9%	25.4%	25.4%
**Recognizing people's faces**	32.8%	29.9%	25.4%	10.4%	1.5%

*Perceived impact by those participants who had a previous pair of reading glasses.

N/A, not applicable.

Finally, the participants were questioned about their perceived value of the present intervention. A bimodal distribution was observed in the participants’ responses when asked about their willingness to pay for the pair of reading glasses they received at the health outreach: 28.4% stated they would be willing to pay >4 USD while 27.7% stated that they would be unable to pay. Considering the progressive nature of presbyopia, the final question posed to the participants dealt with their willingness to participate in a program where their current pair of reading glasses would be exchanged for a new pair in 3–5 years. Over 80% of the participants responded that they would be very likely to participate in such a program. An unexpected finding was that 84.3% (118/140) of the participants stated the reading glasses they received at the health outreach would greatly improve their ability to earn a living.

## Discussion

This study piloted a simple and cost-effective intervention aimed at increasing access to reading glasses in a rural, indigenous, Filipino population. At the same time, the intervention served to increase awareness of presbyopia and assessed the perceived barriers and benefits to accessing treatment for the age-related decrease in the ability of the eye to focus on near objects in this population.

Clinical evidence suggests that the prevalence of uncorrected presbyopia can approach 100% among the elderly population and begins at earlier ages in the setting of developing countries [1,14,15]. The prevalence of presbyopia was not assessed in our population because this was not a population-based study but rather a pilot study whose results may not be generalizable. However, the results of this pilot study do indicate that presbyopia is a common problem within these indigenous communities in the Philippines with a significant unmet need. Furthermore, upon application of ready-made single-vision glasses intended to improve near vision, more than 77% of the participants with near vision impairment corrected to 20/40 or better. This large improvement in near visual acuity highlights the ease to which this condition can be remedied using a simple, cost-effective intervention with limited personnel, training, and time.

With regards to our expedited intervention, which did not take into account best corrected distance vision, a study examining presbyopia in Zanzibar, Africa [5] showed that only 10.2% of its population, on presentation, was able to see 20/50 at 40 cm. Yet, with near add, the proportion of individuals who could see 20/50 near increased to 98.2%. Moreover, correcting for distance visual acuity had little impact on near visual acuity, only increasing this percentage from 10.2% on presentation to 10.8% after distance correction. This finding further justifies our simple, cost-effective intervention, which rapidly dispensed ready-made, single vision glasses to improve binocular near vision.

A recent study assessing presbyopia in a rural, Chinese population (Shenyang, northern China) also utilized a functional definition of presbyopia [16,17] and did not determine distance refractive error. Of those who completed their questionnaire, 69.3% fit their definition of presbyopia (binocular near visual acuity <20/50 at 40 cm with habitually worn distance correction, with improvement in near vision by at least one line of acuity upon application of a plus lens). In comparison, 87.6% of our participants presented with near visual acuity <20/50 that was improved at least one line of acuity upon application ready-made single-vision glasses. The difference in percentages may be due to the fact that our participants were not tested with their habitually worn refractive correction, nor did we exclude any participants with distance vision <20/63 (as in the Chinese study). Considering the Filipino population studied here is a near equatorial population, the negative correlation between the age of onset of presbyopia and average annual temperature [18] may also be playing a role in the difference in percentage of participants presenting with near visual acuity <20/50 between the two studies. However, our pilot study may represent the worst case scenario with regards to near vision impairment because those who presented to the health outreach knew eye care would be provided as well as reading glasses at no charge.

In conjunction with the improvement in near visual acuity, we wanted to ascertain that our pilot intervention was providing functional improvement as well. The study in Zanzibar, Africa [5] assessed improvement via a visual function questionnaire at baseline and at 6-months follow-up as well as with the ability to thread a needle with and without ready-made near spectacles. The universality of this task is questionable and limited by factors other than vision especially prevalent in an aged population, such as arthritis and impaired manual dexterity. With limited personnel and time, a follow-up visual function questionnaire for individual subjects was not possible. Furthermore, we sought a more quantitative, graded measure of functional improvement and chose change in stereoacuity upon the application of reading glasses as a surrogate measure of functional improvement.

Stereopsis is the binocular perception of depth [19], which is necessary for such tasks as threading a needle. Stereoacuity is the smallest difference in depth that can be discriminated with binocular vision [20], and it has been shown to improve with improved near visual acuity [21]. Furthermore, with regards to its functional significance, stereoacuity has been reported to be a significant, independent, risk factor for self-reported visual disability in an elderly population [22]. Stereoacuity has also been associated with the ability to implement a medication regimen [23], and more recently, it was determined that stereoacuity impacted the performance on certain motor skills tasks, such as the Purdue Pegboard test and placing beads on a needle [24]. Thus, the understanding of the functional significance of stereoacuity, the ability of the RANDOT® stereoacuity test to provide quantitative, graded values, and the ease at which this test could be administer made the change in stereoacuity an ideal surrogate measure for functional improvement in this pilot study.

Among those participants that demonstrated stereopsis, more than two-thirds showed an improvement in arc-seconds of resolution of stereoacuity upon the application of reading glasses. Subsequently, it may be suggested that our simple and cost-effective intervention provided functional improvement as well as improvement in near visual acuity. However, ten participants did fail to demonstrate measurable stereoacuity pre- and post-correction and another 16 participants actually showed a small decline in stereoacuity upon the application of reading glasses (Table 2). Although there was no difference in near visual acuity pre-correction, the ten participants that failed to demonstrate any stereoacuity did have worse near visual acuity following near vision correction only achieving, on average, a corrected near visual acuity of <20/50 (p < 0.0001); the average corrected near visual acuity of participants demonstrating stereopsis was >20/30. This finding may suggest a greater severity of ocular comorbidities or a lack of functional binocular vision from a variety of causes (e.g. asymmetric cataract, refractive error, or undiagnosed amblyopia or strabismus) in this subset.

With regards to those who actually showed a decline in the best-measured stereoacuity post-correction, more than half of these individuals actually showed improvement on some portions of the test but not others. Specifically, they showed improvement on the simple shapes and animal portions of the stereotest that contain two and three levels of gross disparity, respectively, but exhibited a decline in measured stereoacuity when tested on the finely graded ten panel sequence (see Methods). Considering stereoacuity was measured immediately after the participants received their reading glasses, relaxation of the accommodation reflex with the addition of plus lenses may be a possible cause for the decline in measured stereoacuity in a small subset of patient with underlying hyperopia. Giving participants time to adapt to the reading glasses could have potentially overcome this effect.

Beyond correcting near visual acuity and providing functional improvement with ready-made near spectacles, we also sought to understand the perceived barriers and benefits to accessing treatment to presbyopia in a predominantly indigenous population. Two different barriers were the focus of our investigation: cost of so-called “reading glasses” and the availability of an eye doctor. Not surprisingly, more participants perceived that the cost of reading glasses greatly prevented them from obtaining access as compared to the availability of an eye doctor (Table 3). Furthermore, the main reason cited for not having a previous pair or reading glasses was lack of money. Although only two physicians that specialize in eye care are registered in the province of Nueva Vizcaya, there are at least 15 optometrists offices or optical shops in the province; eyeglasses can be purchased either at these optical shops or even from vendors in the local market, providing a possible reason why availability was not perceived as a barrier to the same degree as cost. In addition, the average monthly family income is approximately 200 USD [25]. Coupling this income with the fact that it costs greater than 150 USD per month to provide a family of five with basic food and non-food requirements [25] and that a consultation with an eye specialist can cost between 20 and 35 USD in the local market, it is understandable why cost should be perceived to be a greater barrier than availability.

The final aspect of the questionnaire focused on the participants’ perceived value of the present intervention. 28.4% of the participants were willing to pay >4 USD for the current pair of reading glasses, approximately eight times the actual cost of the spectacles provided (0.50 USD), which may be depicting the value the participants placed on the current intervention. However, in agreement with cost as a major barrier to accessing treatment for presbyopia, >40% of the participants stated they could pay <1 USD or nothing at all for the current pair of reading glasses. Hence, a community distribution scheme is needed that is amenable to both subsets of participants. For that reason, a cross-subsidization protocol may not be financially sustainable. Further information, such as the least amount that would be possible for those unable to pay, is necessary before such a protocol can be enacted. Therefore, we proposed a glasses trade-in/recycle program to the participants. In this program, an individual would have the opportunity to trade-in his or her reading glasses for a different pair every three to five years (in accordance with the natural progression of presbyopia), if upon examination, they were deemed to need a different, stronger power. If in useable condition, the spectacles could then be recycled to another individual.

While this pilot study is the first to investigate presbyopia in a rural Filipino population and highlights the possible benefits that can be achieved by the correction of near vision by simple ready-made glasses, it does have its limitations. First, we had a limited sample size (142 participants) in comparison to other studies that have been performed in the developing world [5,7,16,17]. Second, the study described here is not a population-based study, so the prevalence of presbyopia in the rural province of Nueva Vizcaya cannot be determined. Also, by not providing a detailed ophthalmic exam or distance refraction due to limited resources and time, this group of individuals may have unforeseen ocular comorbidities and other refractive errors, in addition to presbyopia. In terms of the survey instrument, we could not completely control for responder bias as the participants knew they would be receiving reading glasses. Yet, to reduce the risk of bias, the participants were given their spectacles before being asked to participate in the survey. Finally, the majority of participants were female. Because this is a pilot study and its results may not be generalizable, it is hard to know exactly how this demographic factor affected the conclusion. Yet, no association was found between the sex of the participants and uncorrected near visual acuity.

## Conclusions

Uncorrected presbyopia is a significant cause of age-related visual impairment in this indigenous Filipino community, and dispensing ready-made single-vision glasses is a simple and cost-effective intervention to improve near vision and enhance depth perception. Furthermore, understanding of the barriers and benefits to correcting near visual acuity will help in the design and execution of sustainable programs to improve access to ready-made spectacles in developing countries. Further research and resources should be directed toward conducting comprehensive population-based intervention studies aimed at developing sustainable programs capable of relieving the unmet global burden of presbyopia.

## Abbreviations

D: Diopter; ANOVA: Analysis of variance; USD: United States Dollar.

## Competing interests

The authors declare that they have no competing interests.

## Authors’ contributions

TJW participated in data collection, data analysis, and drafted the article. CMG played a predominant role in the design of the study. MS participated in the data collection and questionnaire administration. GSW participated in the design of the questionnaire. GPG participated in data collection. DJR played a major role in the design of the study, data analysis, and conceptualization of the article. All authors read and approved the final manuscript.

## Pre-publication history

The pre-publication history for this paper can be accessed here:

http://www.biomedcentral.com/1471-2415/14/9/prepub
